# The Role of α-Linolenic Acid and Its Oxylipins in Human Cardiovascular Diseases

**DOI:** 10.3390/ijms24076110

**Published:** 2023-03-24

**Authors:** Lucia Cambiaggi, Akash Chakravarty, Nazek Noureddine, Martin Hersberger

**Affiliations:** 1 Division of Clinical Chemistry and Biochemistry, Children’s Research Center, University Children’s Hospital Zurich, University of Zurich, 8032 Zurich, Switzerland; 2 Center for Integrative Human Physiology, University of Zurich, 8032 Zurich, Switzerland

**Keywords:** lipid mediators, oxylipins, inflammation, cardiovascular disease, mortality, α-linolenic acid, HOTrE, DiHOTrE, oxo-OTrE, EpODE

## Abstract

α-linolenic acid (ALA) is an essential C-18 n-3 polyunsaturated fatty acid (PUFA), which can be elongated to longer n-3 PUFAs, such as eicosapentaenoic acid (EPA). These long-chain n-3 PUFAs have anti-inflammatory and pro-resolution effects either directly or through their oxylipin metabolites. However, there is evidence that the conversion of ALA to the long-chain PUFAs is limited. On the other hand, there is evidence in humans that supplementation of ALA in the diet is associated with an improved lipid profile, a reduction in the inflammatory biomarker C-reactive protein (CRP) and a reduction in cardiovascular diseases (CVDs) and all-cause mortality. Studies investigating the cellular mechanism for these beneficial effects showed that ALA is metabolized to oxylipins through the Lipoxygenase (LOX), the Cyclooxygenase (COX) and the Cytochrome P450 (CYP450) pathways, leading to hydroperoxy-, epoxy-, mono- and dihydroxylated oxylipins. In several mouse and cell models, it has been shown that ALA and some of its oxylipins, including 9- and 13-hydroxy-octadecatrienoic acids (9-HOTrE and 13-HOTrE), have immunomodulating effects. Taken together, the current literature suggests a beneficial role for diets rich in ALA in human CVDs, however, it is not always clear whether the described effects are attributable to ALA, its oxylipins or other substances present in the supplemented diets.

## 1. Introduction

Humans can synthesize saturated fatty acids, and although they have desaturases to introduce some double bonds, they do not have the desaturases for introducing double bonds at the n-3 and n-6 positions of de novo produced fatty acids. The n-3 fatty acid α-linolenic acid (ALA, C18:3) and the n-6 fatty acid linoleic acid (LA, C18:2) are therefore essential fatty acids for humans and ought to be ingested with food [1]. ALA is abundant in higher plants and algae and is particularly enriched in plant oils such as chia, walnut and flaxseed (linseed), but can also be found in very small amounts in some fish such as wild sardines [2,3]. LA is commonly provided by vegetable oils such as sunflower and corn oil.

In the body, LA is a precursor to arachidonic acid (AA, C20:4), while ALA is a precursor to eicosapentaenoic acid (EPA, C20:5) and docosahexaenoic acid (DHA, C22:6), via a series of desaturation and elongation steps. Although humans can create EPA and DHA from ALA, the conversion rate is low, especially for DHA, and appears to be affected by gender, human genetics and environmental factors. Therefore, dietary supplementation is advised to obtain adequate levels of these long-chain n-3 PUFAs [4,5]. The rate-limiting enzyme for the conversion of ALA to EPA and DHA seems to be the polymorphic delta-6-desaturase and there is an indication that this conversion is reduced by competition with the n-6 PUFA, LA, which is desaturated and elongated by the same enzymes [6,7]. Consequently, the ratio of n-6/n-3 PUFAs in the diet can dictate how much ALA gets converted to EPA and DHA. In support of this, a recent systematic review deduced that it is achievable to increase the EPA and, to some extent, the DHA PUFA status in humans by limiting n-6 LA and/or increasing n-3 ALA intake, although it can be argued that the degree of these changes is considerably less than what can be achieved via dietary supplementation of n-3 long chain PUFAs [7].

There is ample evidence for an anti-inflammatory and pro-resolution effect of the long-chain n-3 PUFAs, EPA and DHA. After they are taken up by the body, they are distributed to different tissues, in the form of triglycerides, where a substantial part will be integrated into the phospholipids of the cell membranes [8,9]. Incorporation of EPA and DHA into cell membrane phospholipids will disrupt membrane rafts [10], and when EPA and DHA are released from the membrane, they suppress inflammatory signaling by activating peroxisome proliferator-activated receptor gamma (PPAR-γ) and free fatty acid receptor 4 (FFA4) [11,12,13]. In addition, in recent years, a new immunomodulatory role for the long-chain n-3 PUFAs has been described, which involves the generation of oxylipins called specialized pro-resolving mediators (SPMs) [11,14]. These long-chain n-3 PUFA-derived oxylipins play an essential role in the resolution of inflammation and their role in inflammation and inflammatory diseases has been the focus of several recent reviews [11,15]. In this review, we will discuss the role of the shortest essential n-3 PUFA in inflammation and cardiovascular diseases (CVDs), the C-18 ALA, by summarizing findings of meta-analyses on epidemiological and randomized controlled trials (RCTs) in CVDs, and by summarizing the mechanistic studies of ALA-derived oxylipins in animal and human cellular models of inflammation.

## 2. ALA Intake, Human Inflammation and CVDs

### 2.1. Association of ALA with the Lipid Profile and Markers of Inflammation in Human Plasma

Altered lipoprotein profiles are risk factors for many diseases [16] and lowering low-density lipoprotein (LDL) cholesterol is a recommendation for primary and secondary prevention of CVDs. Several guidelines for cardiovascular risk reduction recommend a diet rich in long-chain EPA and DHA for cardiovascular risk prevention [17,18] and there is a recommendation to lower plasma triglycerides by pharmacological supplementation of EPA and DHA (2–4 g/day) in patients with CVDs who have their LDL cholesterol lowered by efficient statin therapy but still have hypertriglyceridemia [19].

Hence, the effect of EPA and DHA on the lipoprotein profile has been studied extensively, showing in a meta-analysis of RCTs that EPA and DHA efficiently decrease triglyceride levels, while they scantly increase total LDL and high-density lipoprotein (HDL) cholesterol [20]. In contrast, dietary intake of ALA was shown to scantly reduce the concentrations of triglycerides (weighted mean difference (WMD) −0.101 mmol/L), total cholesterol (WMD −0.140 mmol/L), LDL cholesterol (WMD −0.131 mmol/L) and very low-density lipoprotein (VLDL) cholesterol (WMD −0.121 mmol/L) in a recent meta-analysis by Yue et al. (Table 1) [21]. Investigating the dose- and time-response of ALA on lipid traits, the meta-analysis suggests a maximal effect of 3–8 g/day of dietary ALA after 2–3 months of treatment, with higher doses and longer treatments being less efficient [21]. When Chen et al. compared, in their meta-analysis of RCTs, the triglyceride lowering effect of ALA to the effect of the long-chain PUFAs EPA and DHA, they found that ALA is less efficient than the long-chain PUFAs in reducing triglyceride levels (WMD −0.191 mmol for EPA/DHA) [20]. Subsequent subgroup analyses showed that the lower efficiency of ALA in reducing triglycerides compared to the long-chain PUFAs EPA and DHA was preferentially pronounced in older people (>40 years old) and in individuals with higher baseline triglyceride levels [20]. No effect of ALA was observed on HDL levels [20,21].

Another risk factor for CVD is chronic inflammation, which is associated with higher levels of inflammatory biomarkers such as C reactive protein (CRP) and pro-inflammatory cytokines [28]. A meta-analysis of studies investigating the effect of ALA supplementation on the blood levels of inflammatory biomarkers revealed no evidence of a beneficial effect on the concentrations of inflammatory markers such as tumor necrosis factor alpha (TNF-α), interleukin-6 (IL-6), CRP, soluble intracellular adhesion molecule (sICAM-1) and soluble vascular cell adhesion molecule (sVCAM-1) [22]. This meta-analysis included studies in healthy volunteers and cardiometabolic cohorts and stated a large heterogeneity in the doses and sources of ALA between studies. Nevertheless, they noticed that studies with a high baseline concentration of CRP (>6.0 mg/L) showed a beneficial effect of ALA, stating that an ALA effect might be more evident in subjects with higher baseline concentrations of CRP [22]. This motivated de Abreu et al. to perform another meta-analysis in patients with higher baseline CRP levels, namely patients with chronic kidney disease. In this meta-analysis of patient cohorts with CRP levels ranging, on average, from 2.2 mg/L to 13.4 mg/L, supplementation with ALA reduced CRP levels by 1.3 mg/L, arguing for an anti-inflammatory effect of ALA supplementation in patients with chronic kidney disease [23].

### 2.2. ALA Supplementation and Cardiovascular Diseases

With the possible beneficial effects of ALA on risk factors for CVD, the question arises of whether ALA supplementation will ultimately reduce the risk for CVDs. In this line, there is an indication that higher consumption of omega-3 fatty acids, especially from marine sources, might decrease CVD mortality, although several studies reported conflicting results [29]. A recent meta-regression analysis combining 135,267 participants concluded that supplementation with EPA and DHA is an effective lifestyle strategy for CVD prevention and that the protective effect probably increases with dosage [30]. This led Elagizi et al. to emphasize on the importance of dosage and baseline levels for supplementation of n-3 long-chain PUFAs, suggesting that higher doses of EPA and DHA will be necessary for CVD prevention in the case of low n-3 long-chain PUFA baseline levels, and lower doses are necessary when the baseline levels of EPA and DHA are already high [31].

Individual studies investigating the role of ALA in CVD prevention also produced conflicting results, but the available meta-analyses on observational studies point towards a beneficial effect of ALA in CVDs [24,25,26]. The first meta-analysis by Pan et al. revealed that a higher ALA intake is associated with moderately lower risk for CVD, with a risk ratio (RR) of 0.86 [25], and the latest meta-analysis by Naghshi et al. showed a reduction in all-cause mortality, CVD mortality and coronary heart disease (CHD) mortality by 10%, 8% and 11%, respectively [26]. Support for a cardioprotective effect of ALA comes from a recent large Mendelian randomization study. This study indicated that genetic polymorphisms that were previously associated with higher ALA plasma levels are also associated with lower ischemic heart disease [32], indicating that higher ALA plasma levels are causally related to ischemic heart disease.

The cardioprotective effect of ALA in epidemiological and genetic studies, however, was not fully corroborated by RCTs investigating ALA supplementation in CVDs. A recent meta-analysis on these RCTs concluded that ALA supplementation has little or no influence on CVD, but that ALA slightly reduces CVD events and risk for arrhythmias with low-certainty evidence [27].

This discrepancy between the observational studies and the RCTs may derive from the general challenge of meta-analyses in nutritional studies. These meta-analyses combine studies using different natural sources for ALA supplementation, ranging from nutritional advice to administering concentrated plant oils. Such an approach may, on one hand, mask a positive effect of ALA, when supplementation studies with oils rather low in ALA (such as olive oil) and with high LA/ALA ratios are included. It may, on the other hand, overestimate its effect because other components such as polyphenols and tocopherols, present in walnuts and, for example, flaxseed oil, might contribute to the beneficial effect in nutritional trials [33].

Therefore, several studies were designed to more directly investigate an association of ALA with CVD, investigating the association of tissue ALA levels with mortality and CVDs. A meta-analysis of 15 such observational studies showed an inverse association of tissue ALA levels with all-cause mortality (RR: 0.95) and estimated that each 1 standard deviation increment in blood concentrations of ALA was associated with a 3% lower risk of mortality [26]. However, no association was identified between ALA blood levels and CVD, possibly for the reason of low numbers [26].

In conclusion, there is evidence that food and oils rich in dietary ALA modify the risk for CVDs and that they are generally healthy and reduce overall mortality. It is, however, difficult to illustrate the role of ALA in these beneficial effects because of the presence of other beneficial nutritional components in the ALA-containing supplementation. In addition, there seems to exist a synergistic effect between the long-chain PUFAs EPA and DHA and ALA because, in a recent longitudinal study, the highest reduction in all-cause mortality occurred in participants meeting both the recommendation for daily consumption of EPA and DHA and of ALA [33].

## 3. Enzymatic Metabolism of ALA to Oxylipins

Oxylipins are enzymatic and non-enzymatic oxidation products from PUFAs and, in the case of the enzymatically produced oxylipins, they are bioactive lipid metabolites, which are thought to mediate a major part of PUFAs’ effects in the body [34,35]. These effects include blood pressure regulation and the regulation of immunity, where the oxylipins are produced during the inflammation and the resolution phase [36,37]. The n-3 PUFAs, such as ALA, EPA and DHA, and the n-6 PUFAs, such as arachidonic acid (AA), LA, γ-linolenic acid (GLA), dihomo-γ-linolenic acid (DGLA) and adrenic acid (AdA), serve as precursors for oxylipin formation [34]. As mentioned, dietary intake or supplementation with n-3 PUFAs leads to the incorporation of these n-3 PUFAs into phospholipids, usually at the SN_2_ position [38].

ALA was recently shown to become selectively incorporated at the SN_1_ or the SN_2_ position into different phospholipid classes in liver cells. There was preferential incorporation mainly into some phosphatidylcholines, phosphatidylethanolamines and some phosphatidic acids [39]. Such PUFAs at the SN_2_ position of glycerophospholipids undergo rapid remodeling via alternating phospholipase A_2_-mediated (PLA_2_) cleavage and lysophospholipid acyltransferase mediated re-acylation, maintaining a distinct content and composition of the various individual glycerophospholipids [40]. Such PUFAs at the SN_2_ position of membrane phospholipids can, however, also be released when cytosolic PLA_2_ is activated during inflammation [35]. Cytosolic PLA_2_ preferentially releases AA but also releases, with a much lower efficiency, C-18 PUFAs such as linoleic acid and the monounsaturated fatty acid, oleic acid [41]. The presence of rather large amounts of oxylipins from ALA in stimulated macrophages indicates that ALA is released in sufficient quantities for the production of oxylipins in these cells [42]. We therefore summarize, in this part of the review, the biochemistry of the enzymatic oxylipin generation from ALA to better understand the potential role of these oxylipins in human health and disease.

**Figure 1:**
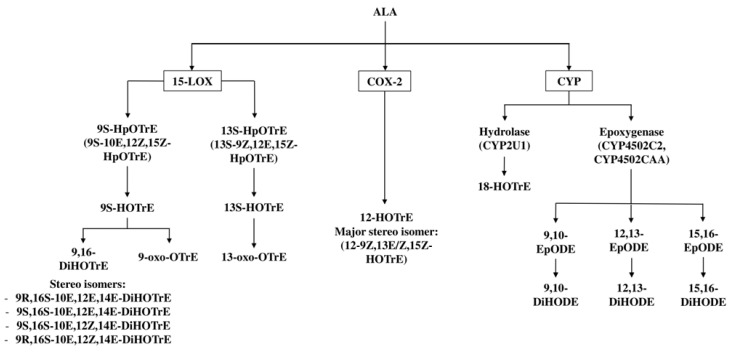
**Schematic illustration of the oxylipins derived from ALA after enzymatic metabolism.** 9S-hydroperoxyoctadecatrienoic acid (9S-HpOTrE) and 13S-hydroperoxyoctadecatrienoic acid (13S-HpOTrE) are produced from ALA via the 15-lipoxygenase (15-LOX) pathway, and are further hydrolyzed to 9S- and 13S-hydroxyoctadecatrienoic acid (9S-HOTrE and 13S-HOTrE), respectively. 9S-HOTrE is further oxidized to 9-oxo-octadecatrienoic acid (9-oxo-OTrE) and further hydroxylated to 9,16-dihydroxyoctadecatrienoic acid (9,16-DiHOTrE), respectively, the 4 stereoisomers of which have also been studied. 12-9Z,13E/Z,15Z-hydroxyoctadecatrienoic acid (12-9Z,13E/Z,15Z—HOTrE) is produced as the major metabolic product of ALA via the cyclooxygenase (COX) pathway, mainly by enzymatic action of the COX-2 enzyme. Cytochrome P450 (CYP) epoxygenase activity through the CYP2 isoforms CYP4502C2 and CYP4502CAA results in the formation of 9,10-, 12,13- and 15,16-epoxyoctadecadienoic acids (9,10-EpODE, 12,13-EpODE and 15,16-EpODE), which are further hydrolyzed to their corresponding dihydroxyoctadecadienoic acids (9,10-DiHODE, 12,13-DiHODE, 15,16-DiHODE) through the soluble epoxide hydrolase enzyme. The CYP450 isoform CYP2U1 metabolizes ALA to produce 18-hydroxyoctadecatrienoic acid (18-HOTrE).

The metabolism of PUFAs into oxylipins occurs by three major enzymatic pathways, i.e., the Lipoxygenase (LOX), the Cyclooxygenase (COX) and the Cytochrome P450 (CYP450) pathways [34] (Figure 1).

In the 1990s, it was shown that ALA is metabolized by different plant LOX enzymes to several oxylipins. Out of the several forms of lipoxygenases, it was shown that potato tuber LOX generated mainly 9S-hydroperoxy-octadeca-10E,12Z,15Z-trienoic acid (9S-HpOTrE) [43] from ALA, while the soybean LOX mainly converted ALA into its 13-oxygenated derivative, 13S-HpOTrE, and, only to a smaller extent, to 9S-HpOTrE [44,45]. These hydroperoxides are reactive and are readily reduced to the hydroxylated derivatives 9S-hydroxy-octadecatrienoic acid (9S-HOTrE) and 13S-HOTrE, respectively. These data indicated that ALA-derived oxylipins are generated in plants, which triggered further investigations with human enzymes to investigate whether human 15-LOX enzymes could also metabolize ALA. These studies revealed that recombinant human 15-LOX-2 converts ALA mainly to 13-HOTrE and only produces small amounts of 9-HOTrE [45]. This 9S-HOTrE, however, was shown to also serve as a substrate for human recombinant 15-LOX-2, leading to the formation of the dihydroxylated ALA metabolite 9,16-dihydroxyoctadecatrienoic acid (9,16-DiHOTrE) [44,45]. The formation of 9,16-DiHOTrE was already reported in 1984 by Feiters et al., but the stereochemistry and the double bond configuration of the asymmetric carbons of the oxylipin was not fully elucidated [46]. A later study identified that 9,16-DiHOTrE consists of four isomers when the dihydroxylated compounds are produced from ALA with recombinant soybean LOX, i.e., 9R,16S-10E,12E,14E-DiHOTrE, 9S,16S-10E,12E,14E-DiHOTrE, 9S,16S-10E,12Z,14E-DiHOTrE and 9R,16S-10E,12Z,14E-DiHOTrE (Figure 1). Similarly, the human enzyme 15-LOX-2 was shown to produce dihydroxylated fatty acids with all trans-9,16-DiHOTrEs being the most prominent isomers; however, the metabolism was markedly less efficient than the metabolism by soybean LOX [45].

The hydroxylated fatty acid metabolites of ALA 9-HOTrE and 13-HOTrE can also be further metabolized to their keto forms, i.e., 9-oxo-10E,12Z,15Z-octadecatrienoic acid (9-oxo-OTrE) and 13-oxo-OTrE (Figure 1). So far, this oxidation of the HOTrEs to the ketones was shown to occur in plants and fungi [47] and was corroborated in vitro using soybean and tomato lipoxygenases for the regioselective oxidation of ALA to 9-HOTrE and 13-HOTrE, followed by oxidation of the hydroxy group to the oxo-group with Bobbit’s reagent [48].

Besides the LOX pathway, it has been reported that the COX pathway [49] also metabolizes ALA to HOTrEs. Odette et al. have shown that the major COX oxygenation product of ALA is 12-HOTrE (Figure 1) when ALA is incubated with a cell extract overexpressing COX-1 and COX-2 [49]. Analyzing the efficiency of 12-HOTrE production from ALA by either COX-1 or COX-2 enzymes individually revealed that COX-1 was inefficient in metabolizing ALA, suggesting that ALA would only serve as a substrate for COX-2 but not for COX-1 in vivo [49].

The third pathway that plays a role in the metabolism of ALA is the CYP450 pathway, which results in epoxygenated oxylipins. In a study to evaluate the epoxidation of C18 unsaturated fatty acids by CYP4502C2 and CYP4502CAA, it was found that both CYP450 isoforms produced three epoxides in equal ratios when ALA was used as substrate (Figure 1). The three epoxides are 9,10-epoxy-octadecadienoic acid (9, 10-EpODE), 12,13-EpODE and 15,16-EpODE [50], and their levels are measurable in human serum [51].

These epoxy-fatty acids are chemically rather stable under physiological pH; however, they are still prone to degradation through the hydrolyzation of the epoxy groups to the corresponding di-hydroxy fatty acids by the soluble epoxide hydrolase enzyme [52,53]. Hence, the concentrations of 9,10-dihydroxyoctadecadienoic acid (9,10-DiHODE), 12,13-DiHODE and 15,16-DiHODE, derived from the corresponding epoxy-fatty acids 9,10-EpODE, 12,13-EpODE and 15,16-EpODE, were also measurable in the serum of men. In this study, 15,16-DiHODE was the major ALA-derived metabolite in the human serum of normolipidemic and hyperlipidemic men, followed by its corresponding epoxide (15,16-EpODE). The levels of 9,10-DiHODE and 12,13-DiHODE were lower [54,55]. There is an indication for another ALA metabolite, 18-HOTrE, which may be produced from ALA via CYP2U1 (Figure 1), although its structure has not been fully elucidated [55].

## 4. Effects and Mechanism of ALA and Its Oxylipins on Inflammation in Mice and Cell Cultures

**Figure 2:**
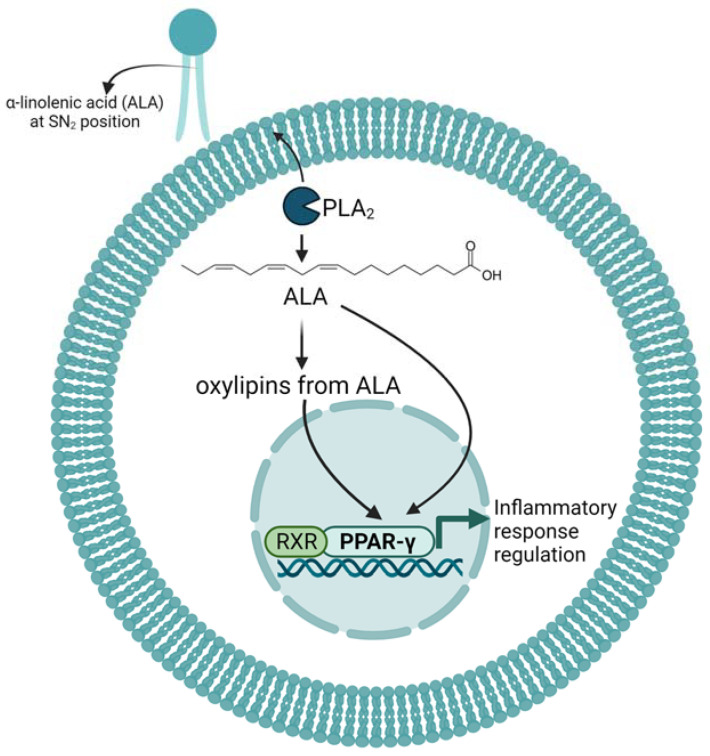
**Schematic summary of the known mechanisms through which n3-PUFA ALA and its oxylipins influence the inflammatory response.** α-linolenic acid (ALA) from diet or supplements incorporates at SN_2_ in cell membrane phospholipids and can be released by phospholipase A_2_ (PLA_2_) upon a cellular signal. ALA can then activate peroxisome proliferator-activated receptor gamma (PPAR-γ) or can be metabolized into oxylipins, which, in turn, can themselves act as ligands for PPAR-γ. PPAR-γ heterodimerizes with the retinoid X receptor (RXR) and this signaling pathway is known to regulate processes of lipid metabolism and inflammation. Created with BioRender.com (accessed on 9 February 2023).

The anti-inflammatory effects of ALA have traditionally been credited to its biological conversion to longer chain n-3 PUFAs, EPA and DHA, which serve as substrates for the synthesis of the potent pro-resolving and anti-inflammatory oxylipins such as resolvins, maresins and protectins [36]. However, as mentioned above, the conversion rate of ALA to EPA and especially to DHA is low [56,57]. Nevertheless, there is an indication that ALA-rich oil has similar immunomodulatory effects to fish oil in mice and cell cultures. For example, ALA supplementation reduced TNF-α production in peritoneal macrophages and in murine whole blood and decreased lymphocyte proliferation in tumor-bearing rats [58,59]. In an in vitro study using RAW 264.7 macrophages, treatment with ALA inhibited gene expression of inflammatory iNOS, COX-2 and TNFα in a dose-dependent manner upon LPS stimulation through the blocking of NF-κB and MAPKs activations [60]. Mechanistically, there is an indication that the reduced pro-inflammatory cytokine production is mediated by PPAR-γ activation [61] (Figure 2). However, it is not clear whether these effects are mediated by ALA or by some of its oxylipin metabolites mentioned above.

Considering that supplementation of ALA does not lead to large increases in mouse tissue [59] and in human red blood cells [62], and that there is only limited conversion of ALA to the longer n-3 PUFAs, the question arises of whether ALA is rapidly metabolized. Indeed, there is an indication that ALA is ß-oxidized at a similar rate as the saturated fatty acid lauric acid, indicating that ALA serves as an energy source [63,64]. However, as outlined above, ALA is also metabolized to oxylipins and these oxylipins are already present in substantial amounts in the oils used for supplementation [65]. Hence, it is not unexpected that mice fed dietary oil sources rich in ALA had rather high concentrations of several ALA oxylipins in blood and tissues [66,67]. This promoted studies focusing on the physiological effects of these ALA oxylipins to answer the question of whether they contribute to the overall anti-inflammatory phenotype associated with ALA supplementation [45].

Limited functional information is available on ALA oxylipins and the role and tissue distribution of most ALA oxylipins, due to a lack of chemically or enzymatically produced standards [15]. However, first-line evidence comes from studies investigating the generation of oxylipins in macrophages. Consecutive treatment of THP-1 macrophages with ALA and lipopolysaccharide (LPS) dampened the secretion of IL-6, TNFα and IL-1β, and this was associated with an increased production of the measured ALA oxylipins, 9-HOTrE, 9-oxo-OTrE, 13-HOTrE and 12,13-EpODE [68]. Interestingly, ALA treatment had a minimal effect on levels of EPA oxylipins and even reduced the DHA oxylipins, which suggests that in this model, the anti-inflammatory effect of ALA does depend on ALA and its oxylipins and not on the oxylipins of the longer chain n-3 PUFAs. However, at the same time, the LOX-derived LA oxylipins 9-hydroxyl-octadecadienoic acid (9-HODE), 9-oxo-octadecadienoic acid (9-oxo-ODE), 13-HODE, 9,10,13-trihydroxy-octadecanoic acid (9,10,13-TriHOME) and 9,12,13-TriHOME were also increased in these LPS-stimulated THP-1 macrophages, indicating a process that favors enzymatic activity towards particular PUFA substrates and not others [68].

Direct support for an anti-inflammatory effect of ALA-derived oxylipins comes from the application of 13-HpOTrE and 13-HOTrE to mouse macrophage cell lines and primary mouse peritoneal macrophages [69]. Specifically, treatment of RAW 264.7 cells with 13-HpOTrE and 13-HOTrE reduced the production of pro-inflammatory markers such as inducible nitric oxide, IL-1β and TNF-α, increased the secretion of the anti-inflammatory IL-10 cytokine and reduced the generation of reactive oxygen species after LPS-stimulation. The anti-inflammatory effect of the two oxylipins was shown to depend on the activation of PPAR-γ, leading to the inactivation of the NOD-, LRR- and pyrin domain-containing protein 3 (NLRP3) inflammasome [69] (Figure 2). Using two different mouse models of sepsis, Kumar et al. extended their investigations and showed that systemic application of 13-HOTrE reduced mortality in both sepsis mouse models. Again, 13-HOTrE reduced the expression of inducible nitric oxide synthase (iNOS) and IL-1β, while it increased the expression of IL-10 in the liver tissue of mice that had septic shock induced by polymicrobial infection [69].

Such an immunomodulatory effect of the HOTREs was also shown in a mouse model of parenteral nutrition, where 9-HOTrE was identified as a major distinction factor between the lipidomes of mice fed a lipid emulsion high in C18 n-3 fatty acids (32% of ALA and stearidonic acid) compared to mice treated with the standard soybean oil emulsion (8% ALA). Mice that received the highly concentrated C18 n-3 fatty acid emulsion showed an overall favorable combination of lower inflammation in the liver, muscle and adipose tissue, with a reduced IL-6 to IL-10 ratio, better insulin sensitivity and an immunity-enhancing phenotype on the gut microbiome [70]. In particular, the gut microbiome analysis revealed that the microinvasive bacterium *A. muciniphila*, intrinsic to mice fed parenteral nutrition [71], was eliminated from the bowel mucosa of mice parenterally treated with highly concentrated C18 n-3 fatty acid emulsions [70]. Intriguingly, supplementation of the soybean emulsion with 9-HOTrE and 13-HOTrE mimicked some of the favorable immune–metabolic phenotypes of the highly concentrated C18 n-3 fatty acid emulsion, thus corroborating that these specific ALA oxylipins may play a role in the immunometabolism in mice [70].

The effects of 9- and 13-HOTrE were also examined on key adipocyte functions that become dysregulated in obesity [72]. Addition of 9- and 13-HOTrE to differentiating 3T3-L1 preadipocytes at a 30 nM dose was enough to reduce triglyceride accumulation in lipid droplets, while higher doses completely blocked lipid accumulation. This was accompanied by decreased intracellular adiponectin, monocyte chemoattractant protein 1 (MCP-1) and TNF-α levels. Comparing the effect of the two HOTrEs revealed that they showed similar efficiencies and that these oxylipins impacted adipocyte function via a posttranscriptional mechanism on proteins involved in lipid metabolism and not by blocking differentiation of preadipocytes to adipocytes. Notably, these effects were also observed after treatment with LOX-derived oxylipins from LA and AA, thus alluding to a similar mechanism of action for all of these oxylipins, which may well be through PPAR-γ activation [73,74,75]. Interestingly, another ALA-derived oxylipin, 13-oxo-OTrE, was also shown to modulate adipocyte function. 13-oxo-OTrE stimulates glucose uptake, adipocyte differentiation and adiponectin secretion in 3T3-L1 adipocytes and there is evidence that these effects are also mediated through PPAR-γ activation [76].

Some mechanistic studies have also been executed with rather high concentrations of the dihydroxylated 15-LOX-derived metabolites of ALA (1–30 µM). Enzymatically produced 9,16-DiHOTrE displayed anti-aggregatory effects on human blood platelets at 1 µM and showed some inhibition of COX-1 and 5-LOX and the resulting prostaglandin and leukotriene production [45]. Intriguingly, these effects were isomer dependent, with the E, Z, E-9,16-di-HOTREs being active. Similarly, a recent study presenting total synthesis of the 9,16-DiHOTREs revealed that 30 µM 9(R),16(R,S)-DiHOTrEs reduces LPS-stimulated secretion of IL-6 and TNF-α in mouse BV2 microglial cells. These rather high concentrations are still 3-fold lower than the concentrations of ALA necessary to elicit similar effects [77].

To our knowledge, no study investigated the physiological effects of the CYP450-derived oxylipins of ALA. There is only evidence that these ALA-derived oxylipins are present in the human bloodstream and that their levels are similar in normolipidemic men and patients with mild combined hyperlipidemia [51]. Most prominent are the epoxide 15,16-EpODE and its hydroxylated product 15,16-DiHODE. This study also showed a positive correlation between ALA levels in human erythrocyte membranes and plasma levels of the ALA-derived free oxylipins 9-HOTrE and 15,16-EpODE, arguing that the ALA oxylipin levels can be directly influenced by diet [51].

## 5. Effect of ALA Supplementation on Oxylipin Profiles

There are conflicting reports on the effect of ALA supplementation on plasma levels of ALA oxylipins. One study in healthy volunteers showed that consuming flaxseed oil supplements increases ALA plasma levels but that it does not influence ALA oxylipin concentrations [78]. On the other hand, two studies in obese women and in hypertensive patients found that ALA supplementation with flaxseed oil increases 9-HOTrE [79,80], and one study indicated that it decreases the levels of CYP450-derived oxylipins from LA and AA [79]. Similarly, supplementation with walnuts (40 g/d for 4 weeks) in hypercholesterolemic women resulted in changes in CYP450-derived oxylipins; however, such walnut supplementation increased plasma levels of the CYP450-derived ALA oxylipins 9(10)-EpODE, 12(13)-EpODE and 15(16)-EpOD, and it also increased the CYP450-derived oxylipins from LA and DHA [81]. These findings might suggest that ALA supplementation leads to higher HOTrE levels only in patients with low level inflammation and that ALA modulates CYP450 metabolism of PUFAs in general.

## 6. Conclusions

There is evidence that oils rich in ALA have beneficial health effects and that these include cardioprotection. Studies investigating the cardioprotective effect revealed that oils rich in ALA scantly reduce the concentrations of triglycerides, total cholesterol, LDL and VLDL, while they have no effect on HDL. In addition, these ALA-rich oils seem to reduce chronic low-grade inflammation in humans, especially in subjects with higher baseline levels of CRP. In line with such a reduction in cardiovascular risk factors, a higher ALA intake is associated with a moderately lower risk for CVD and with a lower risk for all-cause mortality.

Deciphering the mechanisms by which ALA carries out its biological functions is limited by the scarcity of research focusing on ALA, but is also due to the metabolism of ALA to biologically active oxylipins. Such oxylipins are already present in nutritional sources used for ALA supplementation but are also formed enzymatically in mice and men. In particular, the LOX, COX and CYP450 pathways produce oxylipins called HOTrEs, oxoOTrEs, DiHOTREs, EpODEs and DiHODEs.

At least some of these oxylipins seem to play a role in the regulation of inflammation and its resolution. There is good evidence for the two 15-LOX metabolites of ALA, 9- and 13-HOTrE, to display anti-inflammatory and immunomodulatory effects in mice, reducing tissue inflammation and sepsis and improving insulin sensitivity. On a cellular level, 9- and 13-HOTrE reduce the secretion of pro-inflammatory cytokines such as IL-6 and IL-1β and increase the secretion of the anti-inflammatory cytokine IL-10 in mouse monocyte and adipocyte cell lines. These anti-inflammatory effects are, at least in part, mediated through PPAR activation. There is evidence that ALA and some of its oxylipin metabolites activate PPAR-γ, an essential transcription factor in lipid metabolism and inflammation.

## Figures and Tables

**Table 1:** Summary of metanalyses investigating the effect of ALA supplementation.

Study Population	Trials Included	Number of Participants	ALA Source	Dose	Duration	Effects	Reference
At least one of hyperlipidemia, type 2 diabetes, impaired glucose metabolism, hypertriglyceridemia, hypertension, metabolic syndrome (n = 10), healthy subjects (n = 3), unknown (n = 1)	14	1107	Flaxseed oil (n = 6) rapeseed oil (n = 1) linseed oil (n = 2) camelina sativa oil (n = 1) botanical oil (n = 1)	1.9–10 g/d	2–12+ Weeks	↓ LDL	Chen, H. et al. [20]
Healthy (34%), type 2 diabetes (23.4%), dyslipidemia (23.4%), other (19.2%)	47	2630	Flaxseed (46%), walnut (17%), other (37%)	0.4–16 g	3–104 Weeks (mean 15 weeks)	↓ TG, LDL-C, VLDL-C, TC/HDL-C ratio, LDL-C/HDL-C ratio	Yue, H. et al. [21]
Healthy (30%), dyslipidemia (19%), obese (15%), other (36%)	25	2579	Flaxseed oil/linseed (60%) other (40%)	1–14 g/d	4 Weeks-2 years	No CRP reduction	Su, H. et al. [22]
CKD	19	1145	Olive oil (47%), corn oil (21%), flaxseed oil (21%), other (11%)	2–30 g	4–48 Weeks	↓ CRP	de Abreu, A.M. et al. [23]
Unspecified	14	345,202	Unspecified	0.36–2.8 g/d *	4–22 Years follow-up	↓ Risk of composite CHD ↓ risk of fatal CHD	Wei, J. et al. [24]
CVD events	27	251,049	Unspecified	Unspecified	5–30.7 Years	↓ Fatal CHD	Pan, A. et al. [25]
Unspecified	41	1,197,564	Unspecified	0.38–2.69 g/d	2–32 Years follow-up	↓ All-cause mortality, CVD mortality, CHD mortality ↑ cancer mortality	Naghshi, S. et al. [26]
Unspecified	86	162,796	Unspecified	Unspecified	12–88 Months	↓ CVD events ↓ arrhythmia	Abdelhamid, A.S. et al. [27]

* Calculated from percentage of total energy. ↑ increase; ↓ decrease; ALA—α-linolenic acid; CKD—chronic kidney disease; CHD—coronary heart disease; CRP—C-reactive protein; CVD—cardiovascular disease; HDL—high density lipoprotein; HDL-C—HDL cholesterol; IL-6—interleukin 6; LDL—low density lipoprotein; LDL-C—LDL cholesterol; n—number; sICAM-1—soluble intracellular adhesion molecule-1; sVCAM-1—soluble vascular cell adhesion molecule-1; TC—total cholesterol; TG—triglycerides; TNF-α—tumor necrosis factor alpha; VLDL—very low density lipoprotein; VLDL-C—VLDL cholesterol.
